# Evaluation of dentists’ clinical practices and antibiotic use in managing endodontic emergencies in Karachi, Pakistan: a cross-sectional survey

**DOI:** 10.1186/s12903-024-05357-5

**Published:** 2024-12-28

**Authors:** Hassan Yaqoob, Nighat Naved, Shahrukh Ali Khan, Syeda Farah Jabeen, Syed Saqib Raza, Taimur Khalid

**Affiliations:** 1 Department of Surgery, Aga Khan University Hospital, Karachi, Pakistan; 2 Department of Pediatrics, Aga Khan University Hospital, Karachi, Pakistan; 3 Department of Operative Dentistry and Endodontics, Karachi Medical and Dental College, Karachi, Pakistan

**Keywords:** Endodontic emergencies, Antibiotic prescription, Root canal treatment, Antimicrobial resistance, Periapical infection, Pulpitis

## Abstract

**Background:**

Endodontic emergencies, often presented as acute pain or swelling, constitute a substantial challenge in dental practice. While effective management emphasizes prompt intervention, antibiotics are typically indicated only when systemic signs and symptoms are present. There is limited research exists on evaluating the knowledge and clinical approach of dental practitioners in managing endodontic emergencies from our region of the world. Therefore, this study aims to evaluate dentists' knowledge, attitude, and practices regarding clinical management and the prescription of antibiotics in endodontic emergency cases.

**Materials and methods:**

A web-based questionnaire was distributed to working dentists in secondary and tertiary care hospitals and clinics. Eligible participants included dental practitioners, postgraduate trainees, and consultants. The questionnaire included demographic information and inquiries about endodontic emergency treatment approaches, antibiotic prescribing habits, and awareness of guidelines. Statistical analysis was performed Stata software version 17 (StataCorp, College Station, Texas, USA), employing a χ2 test.

**Results:**

Of the 527 dentists approached, 409 completed the survey (response rate: 77.6%). The majority of respondents were female (68%) and general dental practitioners (60.32%) with up to 5 years of experience (80.93%). Survey findings highlighted varied treatment approaches, favoring root canal treatment in multiple visits for cases of both irreversible pulpitis (73.59%) as well as with necrotic pulps/periapical lesions (79.95%). Similarly, 45.48% and 37.9% of the respondents favored pulpectomy combined with analgesics and antibiotics in managing irreversible pulpitis and acute apical periodontitis respectively. Moreover, antibiotics were frequently prescribed for acute apical abscess, with amoxicillin-clavulanate being the most common choice (83.6%). Most practitioners (69%) recommended a minimum of 5-day antibiotic course.

**Conclusions:**

This study highlights Pakistani dentists' preferences in managing endodontic emergencies and prescribing antibiotics. Despite awareness of guidelines and the consequences of overuse, there's a tendency towards antibiotic overprescription, indicating a need for educational interventions to promote rational antibiotic use and mitigate antibiotic resistance.

## Introduction

Endodontic emergencies constitute a significant portion of dental emergency cases, and represent one of the most challenging aspects of clinical dentistry [1]. These emergencies occur when patients experience pain, swelling, or both due to the inflammation or infection of the dental pulp, root canal system, or periapical tissues [2]. The primary goal of managing endodontic emergencies is to alleviate the patient's symptoms, often through immediate intervention [3]. Conditions such as irreversible pulpitis, symptomatic apical periodontitis, and pulpal necrosis are common causes of acute pain and swelling, which necessitate urgent dental treatment [4, 5].

Immediate endodontic intervention is required for the symptomatic relief of these emergencies, although antibiotic prescriptions are only recommended in a minority of cases, specifically those manifesting systemic signs and symptoms [3]. The decision-making process in these cases involves consideration such as whether to perform a single- or multi-visit endodontic treatment, the use of intracanal medicaments, and the inclusion of antibiotics, all of which are guided by established clinical protocols [6, 7]. However, these decisions are also often influenced by the clinician's experience and clinical judgment [8].

Moreover, an important aspect of managing endodontic emergencies involves decision making around antibiotic prescriptions. Current evidence-based guidelines suggest that antibiotics should be reserved for cases with systemic involvement-such as diffuse swelling, fever, or cellulitis—rather than routine endodontic cases [9]. Despite these guidelines, research indicates that antibiotics are often prescribed inappropriately, either in situations where they are not indicated or as a precautionary measure without clear evidence of systemic infection [10]. This over-prescription contributes to the global health issue of antibiotic resistance, a concern that continues to escalate worldwide [11]. Misuse of antibiotics can lead to serious public health consequences, including the proliferation of resistant bacterial strains, longer treatment durations, and higher healthcare costs [12].

Studies conducted in various countries have shown significant variability in dentists’ antibiotic prescription practices for endodontic emergencies [13, 14]. Kaptan et al. examined 589 dentists in Turkey and found that 6.1% would prescribe antibiotics for untreated irreversible pulpitis while 21.7% and 11.4% would prescribe antibiotics for apical periodontitis and acute apical abscesses without any local treatment respectively [15]. According to a survey conducted by Salako et al*.,*90% of Kuwaiti dentists prescribed antibiotics for systemic involvement, whereas only 55% did so for local fluctuant swelling that was not associated with systemic involvement [14].

While some clinicians adhere to established guidelines, others may rely on personal clinical judgment or outdated protocols, which can lead to inconsistent treatment approaches. In Pakistan, few studies have focused on antibiotic prescription practices specifically in the context of endodontic infections however, there is limited research evaluating the knowledge, attitude, and practices of dental practitioners in managing endodontic emergencies [16, 17]. This gap in literature makes it difficult to assess how well dentists are adhering to clinical guidelines or to identify potential areas where antibiotic use could be optimized.

Therefore, this study aims to assess dentists' knowledge, attitudes, and practices concerning the management of endodontic emergencies and the prescription of antibiotics. The focus is to evaluate their adherence to clinical guidelines, identify gaps or inconsistencies in practice, and explore factors influencing their decision-making. By doing so, the study seeks to inform strategies for improving evidence-based practice, enhancing patient outcomes, and supporting global efforts in combating antibiotic resistance through optimized stewardship practices.

## Materials and methods

This was a web-based, cross-sectional analytical survey conducted among dentists of secondary and tertiary care hospitals and private clinics in Karachi, Pakistan, from November 2023 to June 2024. The study protocol was approved as an exemption by the ethical review committee of the Aga Khan University Hospital, Karachi (ERC no. 2023–9266-26864) as no interventions were performed and participation was anonymous. The study was conducted in accordance with the Strengthening the Reporting of Observational Studies in Epidemiology (STROBE) guidelines and the checklist is appended as Supplementary file [18].

According to the eligibility criteria, dental practitioners who were part-time or full-time oral healthcare workers, house officers who had completed their rotation in the department of endodontics, postgraduate trainees, and consultants from all dental specialties were included in the study. Dental students, non-practicing dentists, and practitioners who refused to take part in the study were excluded.

The validated survey instrument employed in this study was adapted from the previously published research conducted by Kaptan et al. [15] The questionnaires were made on Google forms and were electronically distributed to the study participants (527 dentists) whose valid email addresses (sampling frame) were available. Informed consent was obtained at the beginning for participation in the study with the permission to use collected responses for publication purposes. The confidentiality of the data was maintained.

The questionnaire consisted of three parts. The first part comprised demographic information such as age, gender, designation, field of specialization, working institute, years of work experience, and the number of patients seen per day.

The second part included questions regarding the management approaches for irreversible pulpitis, acute apical periodontitis, acute apical abscess, and periapical lesions, including the number of visits for treating vital and non-vital teeth with or without periapical lesions, as well as the use of intracanal medicaments between appointments.

The third section focused on prescribing habits of antibiotics for endodontic conditions, frequently used antibiotics, and scenario-based questions. These questions were adapted from the study by Al-Masan AA et al*. *[19] which explored treatment approaches for various pulpal and periapical conditions. The detailed questionnaire is available in the supplementary file of the manuscript.

Data analysis was conducted using Stata software version 17 (StataCorp, College Station, Texas, USA). Descriptive statistics, including frequencies and proportions, were calculated for qualitative variables such as gender, designation, years of professional experience, field of specialization, and antibiotic prescription habits. To assess the relationship between dentists' knowledge and practices with their years of experience, a chi-square (χ^2^) test was performed, with statistical significance set at *p* < 0.05.

## Results

Amongst the 527 dentists who were asked to participate, 409 completed the survey, resulting in a response rate of 77.6%. The socio-demographic characteristics of the participants are presented in Table 1. The mean age of the participants was 27.6 ± 6 years, with an age range from 22 to 60 years. Among the 409 respondents, most participants were female (*n* = 277/409; 68%) compared to male (*n* = 132/409; 32%). Regarding specialization, (*n* = 250/409; 60.32%) were general dentists, (*n* = 50/409; 12.22%) were endodontists, and the remaining (*n* = 109/409: 26.64%) belonged to other dental specialties. The respondents were also categorized by years of professional experience: up to 5 years, 6–10 years, 11–15 years, and more than 15 years, to facilitate a comprehensive comparison of data. The largest proportion of respondents (*n* = 331/409; 80.93%) reported practicing for 5 years or less.

**Table 1:** Demographic profiles of the participants

Variables	Results
**Age in years (Mean ± S.D)**	27.6 ± 6.09
**Gender**	
Male	132 (32%)
Female	277 (68%)
**Designation**	
Consultant	41 (10.02%)
Post graduate trainees	98 (23.96%)
House officer	144 (35.21%)
General Dental Practitioner	126 (30.81%)
**Professional experience (Clinical)**	
1 – 5 years	331 (80.93%)
6 – 10 years	33 (8.09%)
11−15 years	23 (5.62%)
> 15 years	22 (5.38%)
**Field of specialization**	
General Dentist	250 (60.32%)
Endodontics	50 (12.22%)
Oral and maxillofacial surgeon	53 (12.96%)
Orthodontics	30 (7.33%)
Pediatrics and preventive dentistry	3 (0.73%)
Periodontology	7 (1.71%)
Prosthodontics	16 (3.91%)

### Emergency treatment

For cases of irreversible pulpitis in vital teeth, most dental practitioners preferred multiple-visit root canal treatment (*n* = 301/409; 73.59%). As the practitioners gained more experience, there was a notable shift towards performing the treatment in a single visit rather than multiple visits. Specifically, those with over 15 years of experience performed root canal treatment in a single visit (*n* = 15/22; 68.18%) compared to other groups (*p*-value = < 0.001). For individuals with necrotic pulps and/or periapical lesions, dental practitioners (*n* = 327/409; 79.95%) expressed a preference for performing endodontic treatment over multiple visits rather than opting for a single-visit approach (Table 1). However, this preference did not significantly differ among dentists with varying years of experience (*p*-value = 0.103). The treatment approach, whether single-visit or multiple-visit, also depended on the number of root canals present (Table 2).

**Table 2:** Root canal treatment visits (Multiple vs. Single) in vital and non-vital teeth based on years of experience of dental practitioners

**Number of canals**	**Number of visits**		
	**1 visit**	**2 visits**	**≥ 3 visits**
**One canal**	239 (58.44%)	145 (35.45%)	25 (6.11%)
**Two canals**	142 (34.72%)	177 (43.28%)	90 (22.00%)
**Three canals**	39 (9.53%)	125 (30.56%)	245 (59.90%)
**Four or more canals**	33 (8.06%)	84 (20.53%)	292 (71.39%)

The preferred treatment approach for cases of irreversible pulpitis with normal periapical tissue was pulpectomy combined with analgesics and antibiotics (*n* = 186/409; 45.48%). Importantly, this preference remained consistent across different levels of professional experience. Among practitioners with over 5 years of experience, pulpectomy only emerged as the second most preferred treatment approach. Conversely, practitioners with 5 years of experience opted for pulpectomy along with intracanal medicament as the second most favored approach. Overall utilization of intracanal medication was (*n* = 94/409; 22.98%) among practitioners in cases of irreversible pulpitis (Table 3).

**Table 3:** Distribution of patient visits stratified by the number of root canals. A clear trend is observed where an increase in the number of root canals correlates with a higher number of visits

	**1–5 years**	**6–10 years**	**11- 15 years**	** > 15 years**	**Total**	***p*** **-value**
**Vital teeth**						
Multiple visits	263 (79.46%)	21 (63.64%)	10 (43.48%)	7 (31.82%)	301 (73.59%)	< 0.001
Single visit	68 (20.54%)	12 (36.36%)	13 (56.52%)	15 (68.18%)	108 (26.41%)	
**Non-vital teeth**						
Multiple visits	270 (81.57%)	25 (75.76%)	19 (82.61%)	13 (59.09%)	327 (79.95%)	0.113
Single visit	61 (18.43%)	8 (24.24%)	4 (17.39%)	9 (40.91%)	82 (20.05%)	

In cases of acute apical periodontitis, (*n* = 155/409; 37.9%) of all respondents prescribed analgesics and antibiotics after performing pulpectomy (*p*-value = 0.262) irrespective of years of experience (Table 3). Interestingly, (*n* = 122/409; 29.83%) prescribed analgesics and antibiotics alone, without pulpectomy. The use of intracanal medicament was significantly higher in respondents with experience of 1–5 years (*n* = 64/331; 19.34%).

In cases with acute apical abscess, antibiotic prescription without any invasive treatment was practiced by (*n* = 64/409; 15.65%) of the respondents. However, this number increased to (*n* = 332/409; 81.17%) for antibiotic prescription along with drainage (Table 3). Frequently prescribed antibiotics were amoxicillin-clavulanate (83.6%), metronidazole (62%), amoxicillin (43.1%), cephalosporin (38.2%) and clindamycin (15.4%).

In cases presenting with periapical lesions, root canal treatment emerged as the predominant modality, with (*n* = 205/409; 50.04%) of respondents favoring this approach, irrespective of their years of professional experience (Table 3). Approximately (*n* = 88/409; 21.5%) of participants indicated a preference for performing apical resection in conjunction with root canal treatment, while (*n* = 3; 0.73%) reported sole reliance on apical resection. Extraction was a less common option, chosen by only (*n* = 31/409; 7.58%) of respondents. Moreover, the rate of referrals to endodontists was notably higher (*n* = 82/409; 20%).

### Antibiotic prescription habit

In the surveyed population, amoxicillin-clavulanate emerged as the most frequently prescribed antibiotic for non-allergic patients, with a prevalence of 83.6%. Following closely was metronidazole, prescribed by 62% of respondents. Conversely, in patients allergic to penicillin, the primary choice shifted to cephalosporin, accounting for 48.5% of prescriptions, followed by clindamycin at 42.9%, metronidazole at 30.9%, and erythromycin at 16.4%.

Regarding prescription duration, a substantial majority of respondents, comprising over (*n* = 283/409; 69%) of the sample, advocated for a minimum 5-day course of antibiotics. Only a minority, (*n* = 115/409; 28%), opted for a 3-day regimen, while an even smaller fraction, (*n* = 11/409; 2.69%), recommended 7-day treatment.

When questioned regarding the knowledge of available guidelines and the consequences of overuse of antibiotics, most participants responded affirmatively to both the queries, with (*n* = 264/409; 64.55%) indicating knowledge of guidelines and (*n* = 381/409; 93.15%) acknowledging awareness of the consequences of antibiotic overuse. Among the conditions deemed appropriate for antibiotic prescription by practitioners, cellulitis with systemic complications ranked highest, with 84.2% of respondents considering it suitable for antibiotic treatment. Following closely were acute apical abscesses (71.5%), cellulitis without systemic complications (52%), and symptomatic apical periodontitis (50.2%).

The study participants were presented with six clinical scenarios related to the prescription of antibiotics for various dental conditions, including cases of pulpitis, apical periodontitis, and acute and chronic apical abscesses. In all scenarios, most respondents considered it appropriate to prescribe antibiotics. As per the American Academy of Endodontists guidelines [7], the correct answers and the participants' responses are presented in Tables 4 and 5.

**Table 4:** Emergency treatment approaches for various dental conditions based on years of practitioner experience

	1–5 years	6−10 years	11- 15 years	> 15years	Total	*p*-value
**Emergency treatment approach for pulpitis**						0.012
Analgesic	10 (3.02%)	2 (6.06%)	1 (4.35%)	1 (4.55%)	14 (3.42%)	
Analgesics + Antibiotics	46 (13.9%)	3 (9.09%)	1 (4.35%)	3 (13.64%)	53 (12.96%)	
Pulpectomy	38 (11.48%)	7 (21.21%)	10 (43.48%)	7 (31.82%)	62 (15.16%)	
Pulpectomy + Analgesic + Antibiotics	154 (46.53%)	14 (42.42%)	9 (39.13%)	9 (40.91%)	186 (45.48%)	
Pulpectomy + Intracanal medication	83 (25.08%)	7 (21.21%)	2 (8.7%)	2 (9.09%)	94 (22.98%)	
**Emergency treatment approach for acute apical periodontitis**						0.262
Analgesics	10 (3.02%)	1 (3.03%)	1 (4.35%)	0 (0%)	12 (2.93%)	
Analgesic + Antibiotic	104 (31.42%)	8 (24.24%)	5 (21.74%)	5 (22.73%)	122 (29.83%)	
Pulpectomy	23 (6.95%)	3 (9.09%)	3 (13.04%)	4 (18.18%)	33 (8.07%)	
Pulpectomy + Analgesic + Antibiotics	130 (39.27%)	9 (27.27%)	6 (26.09%)	10 (45.45%)	155 (37.9%)	
Pulpectomy + Intracanal medication	64 (19.34%)	12 (36.36%)	8 (34.78%)	3 (13.64%)	87 (21.27%)	
**Emergency treatment approach for acute apical abscess**						0.103
Analgesic only	9 (2.72%)	0 (0%)	0 (0%)	0 (0%)	9 (2.2%)	
Antibiotics if no drainage	56 (16.92%)	3 (9.09%)	2 (8.7%)	3 (13.64%)	64 (15.65%)	
Drainage + Antibiotic	265 (80.06%)	30 (90.91%)	21 (91.3%)	16 (72.73%)	332 (81.17%)	
Drainage only	0 (0%)	0 (0%)	0 (0%)	1 (4.55%)	1 (0.24%)	
Drainage then Pulpectomy	1 (0.3%)	0 (0%)	0 (0%)	1 (4.55%)	2 (0.49%)	
Root canal treatment	0 (0%)	0 (0%)	0 (0%)	1 (4.55%)	1 (0.24%)	
**Treatment approach for periapical lesion**						0.091
Apical resection	2 (0.6%)	0 (0%)	1 (4.35%)	0 (0%)	3 (0.73%)	
Extraction	29 (8.76%)	2 (6.06%)	0 (0%)	0 (0%)	31 (7.58%)	
Refer to an endodontist	74 (22.36%)	4 (12.12%)	2 (8.7%)	2 (9.09%)	82 (20.05%)	
Root canal treatment	154 (46.25%)	22 (66.67%)	13 (56.52%)	16 (72.73%)	205 (50.04%)	
Root canal treatment + Apical resection	72 (21.75%)	5 (15.15%)	7 (30.43%)	4 (18.18%)	88 (21.52%)

**Table 5:** Clinical scenarios with respective answers according to American Association of Endodontist Guideline. The third column represent responses in which (  ) means antibiotics are indicated and (  ) antibiotics are not indicated

NO.	SCENARIOS	PARTICIPANTS RESPONSES	ANTIBIOTIC INDICATED OR NOT ACCORDING TO AAE GUIDELINES [7]
1	A 23-year-old female patient complains of severe throbbing localized dental pain related to right maxillary first premolar (UR4). She has been feeling feverish since yesterday. On clinical examination there is no swelling or sinus tract. Radiographic examination of UR 4 reveals a small apical radiolucency. The treatment plan was to carry root canal treatment on UR 4, which you started today		**ANTIBIOTICS ARE INIDCATED**
2	A 35-year-old female patient could not sleep for three nights because of painful maxillary right tooth that presents with dull constant severe generalized pain on the maxillary right side. The maxillary right first molar (UR 6) was previously root filled 9 years ago. It is grossly carious, tender and has grade II mobility. Radiographic examination indicates a root filling with voids and short of radiographic apex by 5 mm. She wants to save the tooth and to have root canal re-treatment. The tooth is extremely painful even to a light touch		**ANTIBIOTICS ARE NOT INIDCATED**
3	A 58-year-old male patient, on examination, shows mandibular right 2nd molar (LR 7) and mandibular right 1st molar (LR 6) with large leaking amalgam restorations. The radiographic examination reveals large apical lesions on both teeth. You have advised root canal treatment on LR7 and LR 6. The patient is fit and well. His medical history reveals rheumatic fever 28 years ago		**ANTIBIOTICS ARE NOT INIDCATED**
4	A healthy 27-year-old male patient having a sharp pain that is localized to mandibular left 2nd molar (LL7). Radiographic examination indicates dental caries extending to the pulp chamber with no apical radiographic changes. Partial pulpotomy was advised. The tooth could not be anesthetized after attempting inferior dental nerve block twice, although the patient lips feel numb		**ANTIBIOTICS ARE NOT INIDCATED**
5	A 62-year-old poorly controlled diabetic male patient presents with dull mild discomfort localized to maxillary right canine (UR 3), which is non-tender, non-mobile with pus discharge through a buccal sinus. The treatment plan is to carry out root canal treatment and to avoid extraction		**ANTIBIOTICS ARE NOT INIDCATED**
6	A 25-year-old male patient complains of dull localized pain and swelling associated with deep caries in mandibular right 2nd molar (LR 7). Periapical radiograph shows widening of periodontal ligaments associated with LR7		**ANTIBIOTICS ARE NOT INIDCATED**

## Discussion

The present study aimed to evaluate dentists knowledge and clinical practices in endodontic treatment and antibiotic prescription during emergency cases. Existing literature indicates a paucity of research focusing on the understanding and attitudes of Pakistani dentists in this context [13, 16, 17]. Therefore, our survey was designed to identify the knowledge gap and assess current practices for managing endodontic emergency cases and antibiotic prescriptions to mitigate the inappropriate use of antibiotics and contribute to global efforts to combat antibiotic resistance.

This was a cross-sectional survey engaging experts from various institutions and opting for a web-based platform due to its cost-effectiveness and efficiency in facilitating participation from diverse locations. A comprehensive questionnaire was utilized in this study, consisting of three distinct parts designed to gather detailed information on the participants' demographics, clinical practices, and antibiotic prescribing habits. This structured approach enabled a thorough evaluation of the current knowledge and practices, highlighting areas that require targeted educational interventions to improve clinical outcomes.

The results of the study revealed a preference for multiple-visit root canal treatment among most practitioners for vital teeth with irreversible pulpitis (*n* = 301/409; 73.59%). This is in concordance with the results of some previous studies conducted in the US, UK, Portugal, and Belgium [20–23]. However, a notable shift towards single-visit treatment was observed among more experienced dentists, particularly those with over 15 years of experience (*n* = 15/22; 68.18%). This trend suggests that with increasing experience, dentists may develop greater confidence and proficiency in managing complex cases more efficiently. Conversely, for necrotic pulps and teeth with periapical lesions, the preference for multiple-visit treatment remained predominant (*n* = 327/409; 79.95%). However, as the years of experience increased there was an equal distribution of the preference of the practitioners opting for a single visit versus multi-visit endodontic treatment. This trend may be attributed to the experienced practitioners being confident enough to follow recent evidence which suggests no difference in outcomes of single versus multi-visit endodontic treatment, while those with lesser experience may hesitate to adhere to changing guidelines [24].

The evaluation of dentist’s approach towards irreversible pulpitis with normal apical periodontium revealed a concerning trend regarding the preference for pulpectomy combined with analgesics and antibiotics (*n* = 186/409; 45.48%) as a primary intervention. This approach, which was consistently favored across practitioners regardless of their years of professional experience, deviates from established guidelines that discourage routine antibiotic prescription in the absence of systemic symptoms or periapical pathology. The American Association of Endodontists (AAE) and the European Society of Endodontology (ESE) explicitly recommend that for irreversible pulpitis without systemic involvement, pulpectomy alone, potentially accompanied by analgesics, is sufficient and preferred for pain relief and infection management [7, 25]. Moreover, studies have confirmed that antibiotics do not provide additional benefit in cases where infection is contained within the pulp, with no signs of apical periodontitis or systemic manifestations [26, 27].

The preference for antibiotic use in such cases raises critical concerns about adherence to evidence-based practices and suggests a possible gap in guideline-based decision-making among practitioners, irrespective of experience level. This discrepancy may reflect a lack of confidence in managing irreversible pulpitis through pulpectomy alone. Furthermore, research has shown that over prescription of antibiotic is often influenced by factors unrelated to clinical necessity, such as practitioner’s concern over patient expectations or medicolegal issues [28–30].

Notably, practitioners with more than five years of experience favored pulpectomy alone as their second-most preferred approach, while those with fewer than five years opted for pulpectomy with intracanal medicaments as their secondary choice. The observed overall utilization of intracanal medicaments (*n* = 94/409; 22.98%) may suggest that less experienced practitioners, in particular, are more inclined to rely on adjunctive measures for symptom control, potentially due to a lack of confidence in performing pulpectomy as a standalone treatment. However, the use of intracanal medicaments and antibiotics without indication contradicts best practice guidelines and may lead to inappropriate therapeutic outcomes [7, 25].

In cases of acute apical periodontitis, (155/409; 37.9%) of respondents prescribed analgesics and antibiotics following pulpectomy, irrespective of their years of experience. This uniformity in practice underscores the critical need for guideline adherence across all experience levels. For acute apical abscesses, the combination of antibiotic prescription with drainage was notably high (*n* = 332/409; 81.17%), reflecting a sound clinical approach. However, (*n* = 64/409; 15.65%) of practitioners who prescribed antibiotics without any invasive treatment indicate a gap in adherence to recommended clinical practices, necessitating further education on the appropriate management of such infections.

Regarding antibiotic prescription practices, the preference for amoxicillin-clavulanate (83.6%) and metronidazole (62%) aligns with common clinical practices but raises concerns about potential over-reliance on broad-spectrum antibiotics. For penicillin-allergic patients, the shift to cephalosporin (48.5%), and clindamycin (42.9%) is consistent with current guidelines yet highlights the need for continuous education on alternative antibiotics to prevent resistance development. Moreover, the high level of awareness regarding antibiotic overuse (*n* = 381/409; 93.15%) and the recognition of appropriate conditions for antibiotic prescription, such as cellulitis with systemic complications (84.2%), indicate a foundational understanding of appropriate antibiotic use among practitioners. Nevertheless, the affirmative responses to prescribing antibiotics in all presented clinical scenarios, despite guidelines suggesting otherwise, reveal gaps in guideline adherence and the need for reinforcing evidence-based practices [7].

While this survey presents valuable insights into the current practices of Pakistani dentists regarding endodontic emergencies and antibiotic prescription, it is imperative to acknowledge several limitations inherent in this evaluation. It is important to recognize the risk of response bias which may be because the respondents might have chosen the options that they believe are likely to be true rather than their own opinions or behaviors, Moreover, owing to the voluntary participation, there is a risk of self-selection bias where those with greater knowledge or interest in the subject matter are more likely to respond resulting in an overestimation of the overall knowledge levels within the broader population of dentists. Lastly, the generalizability of the findings is constrained by the purposive sampling of participants used in this study. However, since it was a multi-centered survey, this could somewhat help mitigate the sampling bias, thus improving the generalizability of the results.

As previously noted, this is probably the first survey in Pakistan to evaluate the knowledge, practices, and approach in endodontic emergency cases and antibiotic prescription among dental consultants, postgraduate trainees, general dental practitioners, and house officers. The results of this study offer valuable insights into the knowledge, clinical practices, and antibiotic prescription habits of Pakistani dentists in the context of endodontic emergencies. With a high response rate of 77.6%, the findings provide a robust overview of current practices and highlight areas that require attention and improvement to combat the rising issue of antibiotic resistance.

## Conclusion

This study highlights significant insights into the current practices of Pakistani dentists regarding endodontic emergencies and antibiotic prescription. The findings underscore the critical need for continuous education and the implementation of standardized guidelines to enhance clinical practices and the appropriate use of antibiotics. By addressing these gaps, we can mitigate the misuse of antibiotics and contribute to the global effort to combat antibiotic resistance. Future initiatives should focus on targeted educational programs, particularly for early-career dentists and ongoing monitoring to ensure adherence to best practices in endodontic treatments.

## Recommendations

Here are the recommendations based on the study's findings:

1. Develop educational interventions for early-career dentists to enhance understanding of evidence-based antibiotic guidelines in endodontic emergencies.
2. Organize regular workshops and seminars to encourage adherence to clinical guidelines for managing endodontic emergencies, reinforcing appropriate antibiotic use.
3. Establish a system to monitor and evaluate antibiotic prescription practices continuously.
4. Conduct further research to assess the impact of educational interventions and refine strategies for judicious antibiotic use.

## Data Availability

The datasets used and/or analysed during the present study are available from the corresponding author upon reasonable request.
